# Level of knowledge of nurses and healthcare technicians regarding soft skills: An exploratory study

**DOI:** 10.1590/1980-220X-REEUSP-2024-0124en

**Published:** 2024-11-11

**Authors:** Jaouad Elkhalladi, Amal Sefrioui, Mohamed El Fahssi, Maroine Tahiri

**Affiliations:** 1 Université Mohamed V, Faculté de Médecine et de Pharmacie, Laboratoire de Biologie Orale et Biotechnologie, Rabat, Maroc.; 2 Université Mohamed V, Faculté de Médecine Dentaire, Laboratoire de Biologie Orale et Biotechnologie, Rabat, Maroc.; 3 Université Mohamed V, Faculté de Médecine et de Pharmacie, Imagerie hybride et radioprotection, Rabat, Maroc.; 4 Université Hassan premier, Institut Supérieur des Sciences de la Santé, Laboratoire des Sciences de la Santé et Technologies, Settat, Maroc.

**Keywords:** Health Sciences, Nursing, Organization and Administration, Communication, Knowledge, Ciências da Saúde, Enfermagem, Organização e Administração, Comunicação, Conhecimento, Ciencias de la Salud, Enfermería, Organización y Administración, Comunicación, Conocimiento

## Abstract

**Objective:**

To assess the knowledge level of nurses and healthcare technicians regarding soft skills (SS) and to identify the most crucial SS.

**Method:**

This is a quantitative exploratory study with an analytical focus, encompassing 350 nurses and healthcare technicians in the Souss-Massa region.

**Results:**

Approximately half of participants (49.7%) exhibited some understanding of SS, with only 12.3% having received any training on the subject. According to participants, the top 10 SS are communication, teamwork, stress management, problem-solving, conflict management, honesty, decision-making, adaptability, lifelong learning, and confidence. Additionally, the prediction model indicates a significant association between SS knowledge and experience (p < 0.001), academic level (p < 0.001), and SS training (p < 0.001).

**Conclusion:**

Nurses and healthcare technicians possess knowledge of SS despite the absence of formal training. Identifying the most important SS in this field is, therefore, invaluable for integration into training programmes for both healthcare professionals and students. Furthermore, additional studies are recommended on SS development and assessment.

## RESUMO

**Objetivo:**

Avaliar o nível de conhecimentos dos enfermeiros e técnicos de saúde relativamente às habilidades comportamentais (HC) e identificar as HC mais importantes.

**Método:**

Trata-se de estudo exploratório quantitativo com enfoque analítico, abrangendo 350 enfermeiros e técnicos de saúde da região de Souss-Massa.

**Resultados:**

Aproximadamente metade dos participantes (49,7%) demonstrou ter algum conhecimento sobre as HC, sendo que apenas 12,3% receberam alguma formação sobre o assunto. De acordo com os participantes, as 10 principais HC são a comunicação, o trabalho em equipa, a gestão do stress, a resolução de problemas, a gestão de conflitos, a honestidade, a tomada de decisões, a adaptabilidade, a aprendizagem ao longo da vida e a confiança. Adicionalmente, o modelo de previsão indica uma associação significativa entre o conhecimento e a experiência sobre HC (p < 0,001), o nível académico (p < 0,001) e a formação em HC (p < 0,001).

**Conclusão:**

Os enfermeiros e técnicos de saúde possuem conhecimentos sobre HC, apesar da ausência de formação formal. A identificação das HC mais importantes neste domínio é, portanto, de grande valor para a integração em programas de formação de profissionais e estudantes de saúde. Além disso, recomenda-se a realização de estudos adicionais sobre o desenvolvimento e a avaliação das HC.

## RESUMEN

**Objetivo:**

Evaluar el nivel de conocimientos de enfermeros y de los técnicos sanitarios en materia de habilidades de comportamiento (HC) e identificar las HC más importantes.

**Método:**

Se trata de un estudio cuantitativo exploratorio con enfoque analítico, que abarca 350 enfermeros y técnicos sanitarios de la región de Souss-Massa.

**Resultados:**

Aproximadamente la mitad de los participantes (49,7%) mostraron algún conocimiento de las HC, y sólo el 12,3% habían recibido alguna formación sobre el tema. Según los participantes, las 10 principales HC son la comunicación, el trabajo en equipo, la gestión del estrés, la resolución de problemas, la gestión de conflictos, la honradez, la toma de decisiones, la adaptabilidad, el aprendizaje permanente y la confianza. Además, el modelo de predicción indica una asociación significativa entre el conocimiento y la experiencia en HC (p < 0,001), el nivel académico (p < 0,001) y la formación en HC (p < 0,001).

**Conclusiones:**

Los enfermeros y los técnicos sanitarios poseen conocimientos sobre las HC a pesar de la ausencia de formación reglada. La identificación de las HC más importantes en este campo es, por tanto, de gran valor para su integración en los programas de formación tanto de profesionales sanitarios como de estudiantes. Además, se recomienda realizar estudios adicionales sobre el desarrollo y la evaluación de las HC.

## INTRODUCTION

The workload, shortage of human resources, epidemics, and an increase in pathologies have heightened burnout and stress among nurses^(1)^. Technological development and the rising demands of the population concerning the quality of care have underscored the necessity for nurses to cultivate and master a spectrum of soft skills (SS)^(2)^. These SS, also referred to as human or socio-emotional skills, serve as excellent indicators of employability and work performance^(3)^.

Numerous studies have demonstrated the utility of SS in healthcare, particularly in organisational and human aspects of patient care ^(4)^. Nurses must remain attentive and prepared to attend to patients, their families, and the wider community. Their contribution to society’s health and well-being relies on their utilisation of skills and experience. Therefore, healthcare professionals must possess SS to meet the diverse needs of patients and the population as a whole^(5)^. The development of SS fosters motivation, optimism, curiosity, and responsibility, helping to mitigate anxiety, work pressure, and demoralisation^(6)^. Additionally, SS promote professionalism, coordination, conviviality, optimism, and confidence, while also facilitating the development of essential skills such as communication, team spirit, problem-solving, and critical thinking^(7)^.

Moreover, the technical and disciplinary knowledge acquired is insufficient for professional conduct in real clinical situations and care activities. Health professionals are expected not only to possess technical skills for enhancing health and treating illnesses but also to exhibit relational and communicational skills for establishing appropriate relationships and effective communication with patients, their families, and all internal and external stakeholders in the hospital structure^(8)^. These SS or personality traits are equally vital for nurse managers in organising, coordinating, and managing nursing care services^(9)^.

Diverse teaching and learning strategies are used to develop SS in nursing students (e.g., simulation and action and blended learning)^(10)^. The latter strategy forms the basis of the flipped classroom, which is a hybrid teaching strategy that permits more interaction and collaboration between students and teachers and among the students themselves, both inside and outside the classroom^(11)^. Additionally, sharing documentation with students before the session through digital platforms allows more time in the classroom.

Few studies have explored SS knowledge among healthcare professionals and its association with socio-demographic variables, such as age, experience, gender, and academic level. Recognising the importance of SS in healthcare, especially in the field of care, and understanding their significance in personal and professional development, this study aims to assess the knowledge level of nurses and health technicians regarding SS and identify the most crucial SS in the realm of nursing and health techniques.

## METHOD

### Design of the Study

This is a quantitative cross-sectional study with an analytical aim, involving the exploration of practitioners’ knowledge of specific SS and the identification of the most important SS according to nurses and health technicians in the Souss Massa region. The study was conducted over a period of 3 months, from November 2022–January 2023, adhering to the STROBE guidelines for cross-sectional studies.

### Population

The study included 2221 nurses and technicians from the nursing and technical health professions practising in the Souss Massa region, encompassing six provinces: Agadir, Inzgane, Tiznit, Taroudant, Chtouka, and Tata. The choice of this region was justified by the accessibility of information and the collaboration of participants within the services and provinces of this region.

### Sampling

The sample size was calculated using the formula:


$$n=t^{2}\times p\times (1-p)/m^{2}$$

– n: Minimum sample size required to obtain significant results for a given event and risk level.
– t: confidence level (the standard value for the 95% confidence level is 1.96).
– p: estimated proportion of the population with the characteristic.
– m: margin of error (generally set at 5%).

Therefore, the minimum desired sample size is 328 with a margin of error of 5% as against 350 participants in this study.

#### Inclusion and exclusion criteria

Inclusion:

- Should be a nurse or health technician.
- Should practise in the Souss Massa region.

Exclusion:

- All other health professionals (doctors, pharmacists, etc.).
- Those practising in other regions of the country.
- Those practising in the private sector.

### Data Collection Tool

The questionnaire was deemed the most suitable tool, allowing for a sufficiently distanced epistemological perspective and ensuring anonymous responses. Comprising three main parts, socio-demographic characteristics, knowledge of SS, and identification of the most important SS, the questionnaire included closed questions, with opportunities for comments. Preliminary testing involved six participants, and 11 incomplete questionnaires were eliminated, leaving 350 for analysis.

### Variables

Nurses are health professionals responsible for providing care, thus ensuring patient satisfaction and quality of care.

Health technicians are professionals who are part of the paramedical staff as laboratory and radiology technicians; they also participate in responding to the needs of patients and the well-being of the population.

SS are non-technical that are crucial in the health sector, especially for personal development and professional success. A non-exhaustive list of 37 soft skills used in nursing and health techniques.

Additional variables, such as age, gender, experience, academic level, field of study, and province, were also included.

### Data Management and Analysis

IBM SPSS Statistics, version 25, was used for data analysis.

Bivariate analysis employed the chi-square test for the relationship between two qualitative variables and the Fisher test for observations with a theoretical size of less than five. Multivariate analysis used the binary logistic regression test to predict the probability of an event occurring. Logistic regression involves testing a regression model in which the dependent variable is dichotomous and the independent variables can be continuous or categorical^(12)^. Variables with a significant relationship (p < 0.05) with the dependent variable in bivariate analysis were included in the multivariate regression model.

### Ethical Aspects

This study received approval from the ethics committee of Mohamed V University in Rabat (no. 39/22). Participation was voluntary, and participants were informed of the study’s purpose and their right to withdraw without any justification. Free, informed, and written consent was obtained before the survey commenced.

## RESULTS

### Socio-Demographic Characteristics

Of our sample, 61.1% participants were women, 82% were aged between 20 and 40, and 60% had more than 6 years of experience. Regarding academic qualifications, the majority (83.7%) held a bachelor’s degree, followed by a master’s degree (8.9%). Furthermore, 46.9% were in nursing, with 29.4% in health techniques. Workplace distribution indicated that 34.9% operated in the Agadir Province, followed by the Inzgane Province (20.9%).

### Knowledge of SS

Nearly half (49.7%) of the participants demonstrated knowledge of SS, with a mere 12.3% having received formal training. Of those trained, 45.24% underwent self-training and 28.6% pursued continuous education, averaging m = 15.79 ± 21.99 hours. In defining SS, 32.3% proposed relational and/or social skills, followed by human skills (25.8%) and non-technical skills (23.6%). Participants prioritised the improvement of working conditions (26.4%), human skills development (25.7%), and patient care (23.6%). The study revealed that 73.4% of participants sought SS training, with 34.9% advocating continuous training.

### The Most Important SS

**Figure 1:**
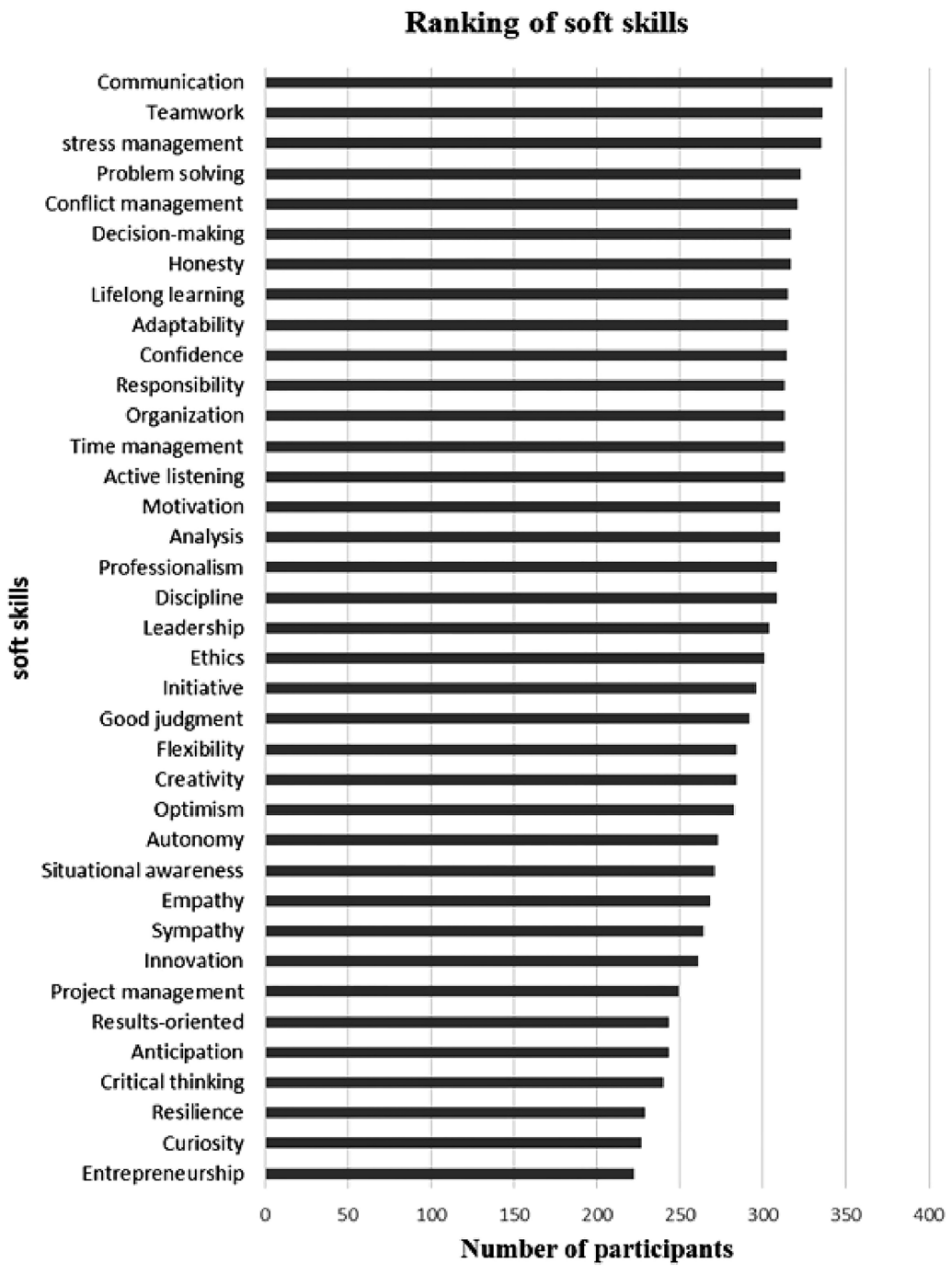
The most important SS.

Figure 1 displays the participants’ ranking of the 10 most crucial SS: communication, teamwork, stress management, problem-solving, conflict management, honesty, decision-making, adaptability, lifelong learning, and confidence.

### Factors Influencing Participants’ Knowledge of SS

Significant relationships were found between knowledge of SS and age (p = 0.004), experience (p < 0.001), academic level (F < 0.001), training in SS (p < 0.001), and the need for SS training (p = 0.018). No significant relationships were observed with gender (p = 0.105), branch of study (p = 0.869), and province (p = 0.156) (Table 1).

**Table 1:** Bivariate analysis of SS knowledge.

**Variables**		**Knowledge of SS**		**p-value**	
		**Yes**	**No**	**KHI2 Test**	**Fisher Test**
Gender				0.105	–
	Male	75(43.1%)	61(34.7%)		
	Female	99(56.9%)	115(65.3%)		
Age (years)				0.004*	–
	Under 40	153(87.9%)	134(76.1%)		
	Over 41	21(12.1%)	42(23.9%)		
Experience (years)				p < 0.001*	–
	Less than 5	89(51.1%)	51(29.0%)		
	More than 6	85(48.9%)	125(71.0%)		
Academic level				–	p < 0.001*
	Bac	2(1.1%)	11(6.3%)		
	Bac+2	3(1.7%)	5(2.8%)		
	bachelor	140(80.5%)	153(86.9%)		
	Master	27(15.5%)	4(2.3% )		
	Doctorate	1(0.6%)	0(0.0%)		
	Other	1(0.6%)	3(1.7%)		
Branch of study				0.869	–
	Nursing	82(47.1%)	82(46.6%)		
	Health Techniques	52(29.9%)	51(29.0%)		
	Midwifery	15(8.6%)	21(11.9%)		
	Social workers	6(3.4%)	6(3.4%)		
	Rehabilitation	19(10.9%)	16(9.1%)		
Province				0.156	–
	Agadir	67(38.5%)	55(31.3%)		
	Inzgane	29(16.7%	44(25.0%)		
	Taroudant	17(9.8%)	26(14.8%)		
	Tata	15(8.6%)	16(9.1%)		
	Chtouka	28(16.1%)	19(10.8%)		
	Tiznit	18(10.3%)	16(9.1%)		
SS Training				p < 0.001*	–
	Yes	41(23.6%)	2(1.1%)		
	No	133(76.4%)	174(98.9%)		
Need training in SS				0.018*	–
	Yes	137(78.7%)	120(68.2%)		
	No	11(6.3%)	27(15.3%)		
	Partially	26(14.9%)	29(16.5%)		

Note. *p < 0.05; SS: soft skills; FC: flipped classroom; ISPITS: higher institute of nursing professions and health techniques.

Table 2 reveals that knowledge of SS is significantly associated with experience (p < 0.001), a master’s degree (p = 0.001), and training in SS (p <0.001).

**Table 2:** Multivariate analysis of SS knowledge.

	**B**	**Signification**	**OR**	**Confidence interval 95%**		
				**Lower**	**Upper**
Experience	–0.987	p < 0.001	0.373	0.222	0.626
Academic level (Master)	3.312	0.001	27.451	3.602	209.188
SS Training	3.231	p < 0.001	25.293	5.663	112.967
Constant	–0.985	0.276	0.374		

*Note*. B: Unstandardized regression weight; FC: Flipped classrooms; OR: Odds Ratio; SS: Soft skills.

## DISCUSSION

The primary objective of this study was to assess the knowledge level of nurses and healthcare technicians regarding SS and identify the most crucial SS.

The participants’ limited understanding of SS (49.7%) can be attributed to inadequate training, as only 12.3% had received training in SS. The SS most commonly identified by participants were socio-relational skills, followed by human skills and then SS, aligning with previous studies along similar lines^(13,14)^. Participants prioritised the importance of SS, with an emphasis on improving working conditions, developing human skills, and enhancing patient care. Human skills were deemed particularly valuable for fostering positive relations and effective communication with patients and their families^(15)^.

Additionally, 73.4% of participants expressed a need for SS training, advocating continuous, self, and basic training as primary methods for SS development. In line with this, Dube and Laari highlighted the increasing demand for SS training driven by factors such as globalisation and population growth, aiming to improve care quality and enhance relationships with patients^(16)^. The critical role of training in SS development, whether through self-training, continuous education, or integrating SS modules into nursing and health technology training programmes, was emphasised.

Moreover, the findings of this investigation identified the paramount significance of 10 specific SS: communication, teamwork, stress management, problem-solving, conflict management, honesty, decision-making, adaptability, lifelong learning, and confidence. The vital role of communication^(17)^, teamwork^(18)^, stress management^(19)^, problem solving^(20)^, conflict management^(21)^, honesty^(22)^, decision-making^(23)^, adaptability^(24)^, lifelong learning^(25)^, and confidence^(26)^, has been consistently recognised in various studies across the literature.

The presence of a significant relationship between SS knowledge and various factors such as age, experience, academic level, SS training, and the perceived need for SS training was observed^(27)^. Multivariate analysis indicated a significant predictive model, where experience demonstrated a notable effect on SS knowledge (p < 0.001 and OR = 0.373). This implies that participants with less than 5 years of experience exhibited higher SS knowledge, possibly influenced by recent government discourse emphasising SS in training and education. A similar observation was made for academic level (master’s degree) with p = 0.001 and OR = 27.451. SS training also significantly influenced SS knowledge (p < 0.001 and OR = 25.293), highlighting the multiplier effect of training on the likelihood of possessing SS knowledge, consistent with previous studies emphasising the usefulness of training for SS development^(28,29)^.

Indeed, in the nursing and healthcare professions, integrating soft skills with basic skills is critical for comprehensive and effective patient care. Soft skills are essential for enhancing the application of technical and clinical competencies (basic skills) required for healthcare tasks. Communication skills are paramount, enabling healthcare professionals to effectively convey information, educate patients, and collaborate with colleagues. Clear communication minimizes misunderstandings and errors, fostering better patient outcomes. Confidence, empathy, and honesty allow healthcare providers to connect with patients on an emotional level. This builds trust and ensuring that patients feel heard and valued, which can significantly enhance the patient experience.

Teamwork is another crucial soft skill, which promoted collaboration among multidisciplinary teams. Effective teamwork ensures that all aspects of patient care are coordinated and optimized, reducing the likelihood of errors and improving overall quality. Problem-solving skills, stress management, adaptability and conflict management empower healthcare professionals to make quick, informed decisions, particularly in high-pressure situations. This ensures patient safety and timely interventions under the best working conditions.

Lifelong learning is also very crucial for developing health professionals’ skills and to maintain technological development in the field. This is also strongly linked to the evolution of nursing practice, the profession’s development, and care quality, and consequently society’s health and well-being.

Training plays an important role in the development of SS. A quasi-experimental study carried out in a Moroccan training institute for nursing professions and health techniques showed that the flipped classroom is capable of developing students’ SS such as adaptation, decision-making, lifelong learning, stress management, communication, and teamwork^(30)^. These skills are ranked among the 10 most important skills according to the participants in our study; hence, it could be beneficial to employ the flipped classroom in the initial training of students in this field. In the same context, continuous training can be useful for developing SS, enhancing motivation, and improving working conditions^(31)^. Also, a soft skills module should be included in the initial training programme for nursing and health technology students.

Encourage health establishments to develop skills training programmes that meet the needs of professionals; incorporate this training into regular staff appraisals. To maintain their relevance and effectiveness over time, promote a culture of continuous improvement by suggesting regular revisions and updates to training programmes based on feedback and new research findings. Exploit the potential of e-learning platforms and mobile applications for flexible and accessible training.

### Limitations

Although this study contributes to understanding nurses’ and healthcare technicians’ knowledge of SS, producing robust results through quantitative approaches and the significant sample size, certain limitations should be acknowledged. It focused solely on the public sector, and professionals in the private sector may have different knowledge levels, particularly concerning working conditions, potentially influencing the relative importance of SS in this sector. Additionally, being confined to the Souss Massa region limits the generalisability of findings to other regions of Morocco or different countries. In this sense, including more diversified samples and qualitative methods in future research may be very beneficial to research robustness.

## CONCLUSION

This study investigated the level of knowledge of SS among nurses and health technicians and the prioritisation of key SS in the field of nursing and health techniques. Despite nearly half of the participants having some understanding of SS, only 12.3% received formal training in the subject. The identified top 10 SS encompass communication, teamwork, stress management, problem-solving, conflict management, honesty, decision-making, adaptability, lifelong learning, and confidence. The predictive model highlights that SS knowledge correlates significantly with experience (p < 0.001), an academic level (p = 0.001), and having undergone SS training (p < 0.001). Consequently, integrating these crucial SS into training programmes for healthcare professionals and students is recommended.

Assessing the level of knowledge of nurses and healthcare technicians regarding SS is a key factor in their development and fulfilment in a dynamic and constantly changing working environment.

Additionally, these findings serve as a valuable database for health professionals, educators, healthcare establishments, and training institutes, fostering the development of health professionals’ skills and consequently enhancing the quality of services and the well-being of patients and the general population. Moreover, further research is warranted to explore the development and evaluation of SS, not only for nurses but also for all healthcare professionals in this region and other areas of the country. Thus, it is important to experiment with several pedagogical strategies to determine those that excel for the development of SS, both for students in initial training and healthcare professionals through continuous training.
